# Effect modification of the association between vitamin B2 intake and diabetes mellitus by sex: findings from the National Health and Nutrition Examination Survey 2013–2020

**DOI:** 10.3389/fnut.2024.1510096

**Published:** 2024-12-16

**Authors:** Hui Yang, Xudong Wang, Xiaolan Xi, Yujia Xia, Mingxia Jiang, Hui Zuo

**Affiliations:** ^1^School of Public Health, Suzhou Medical College of Soochow University, Suzhou, China; ^2^Department of Clinical Nutrition, The Fourth Affiliated Hospital of Soochow University, Suzhou, China; ^3^Jiangsu Key Laboratory of Preventive and Translational Medicine for Major Chronic Non-communicable Diseases, Suzhou Medical College of Soochow University, Suzhou, China; ^4^MOE Key Laboratory of Geriatric Diseases and Immunology, Suzhou Medical College of Soochow University, Suzhou, China

**Keywords:** vitamin B2, diabetes mellitus, NHANES, sex, interaction

## Abstract

**Background and objectives:**

The relationship between vitamin intake and diabetes mellitus (DM) has attracted growing attention. Only few studies have linked vitamin B2 (VB2) and development of DM. In this study, we aimed to assess the association between VB2 intake and DM among U.S. adults.

**Methods:**

We performed a cross-sectional analysis by using four waves of the National Health and Nutritional Examination Surveys (NHANES) data in 2013–2020. A total of 18,338 participants aged ≥18 years were included. VB2 intake was estimated by 24-h dietary recall on the first day. We used multiple logistic regression analysis to assess the association between VB2 intake and DM in men and women, separately.

**Results:**

VB2 intake was significantly associated with DM in women but not in men (*P*-interaction < 0.05). In women, the multivariable-adjusted odds ratio (OR) for the fourth compared with the first quartile of VB2 intake was 0.67 (95% CI: 0.48, 0.93, *P*-trend = 0.025). Each standard deviation increment of log-transformed VB2 intake was associated with 19% reduced odds of DM (*P* = 0.005). In contrast, no significant association between VB2 intake and DM was observed in men (*P*-trend > 0.05). An inverse dose-response relationship between VB2 intake and DM was observed in women, but not in men.

**Conclusions:**

Increased VB2 intake was associated with lower odds of DM in women, but not men. Our study underscores the potential role of VB2 in the prevention of DM in women. Prospective studies from different populations are warranted to confirm our findings.

## 1 Introduction

Diabetes mellitus (DM) has become one of the leading causes of death globally, with 5.2 million deaths from DM worldwide, and a mortality rate of 82.4 per 100,000 (1, 2). It is a metabolic disease that affects people of all geographic regions worldwide (3, 4).

A growing body of evidence has linked vitamins with diabetes. For example, vitamin A and vitamin D have been shown as protective factors for diabetes (5–7). Plasma levels of almost all B vitamins were lower in patients with DM than others (8, 9).

Vitamin B2 (VB2), also known as riboflavin, can be found in a wide variety of foods, especially milk, fruit, vegetables and animal offal (10, 11). It has antioxidant, anti-aging, anti-inflammatory, anti-damage and anti-cancer properties (12, 13). VB2-deficient diet could elevate blood glucose levels in mice (14). In a cellular experiment, it showed that riboflavin insufficiency induced functional changes in adipocytes leading to intensification of their pro-inflammatory, pro-insulin resistance (15). According to a cohort study from Shanghai, China, dietary B vitamin (B1, B2, B6, B9, B12) intake was inversely associated with risk of type 2 DM (16). A recent study reported that higher intake of VB2, especially from food, may be associated with a modestly lower risk of type 2 DM (17). However, this study was based on socioeconomically homogeneous population, and therefore external validity was unknown. Moreover, a Japanese study reported that VB2 intake was inversely associated with DM only in women (18). Interestingly, studies on VB2 in association with other conditions also suggest that VB2 may benefit only in women. For example, a study by Hou et al. reported that VB2 was inversely associated with glaucoma in women, but not in men (19). Another cross-sectional study also showed an inverse correlation between VB2 and early onset sarcopenia only in women (20). Inconsistent findings of VB2 and DM warrants more studies, especially in multi-ethnic populations.

Therefore, we aimed to evaluate the association between dietary and supplement intake of VB2 and DM and examine the potential effect modification by sex, using data of the National Health and Nutrition Examination Survey (NHANES) 2013–2020.

## 2 Methods and materials

### 2.1 Study population

**Figure 1:**
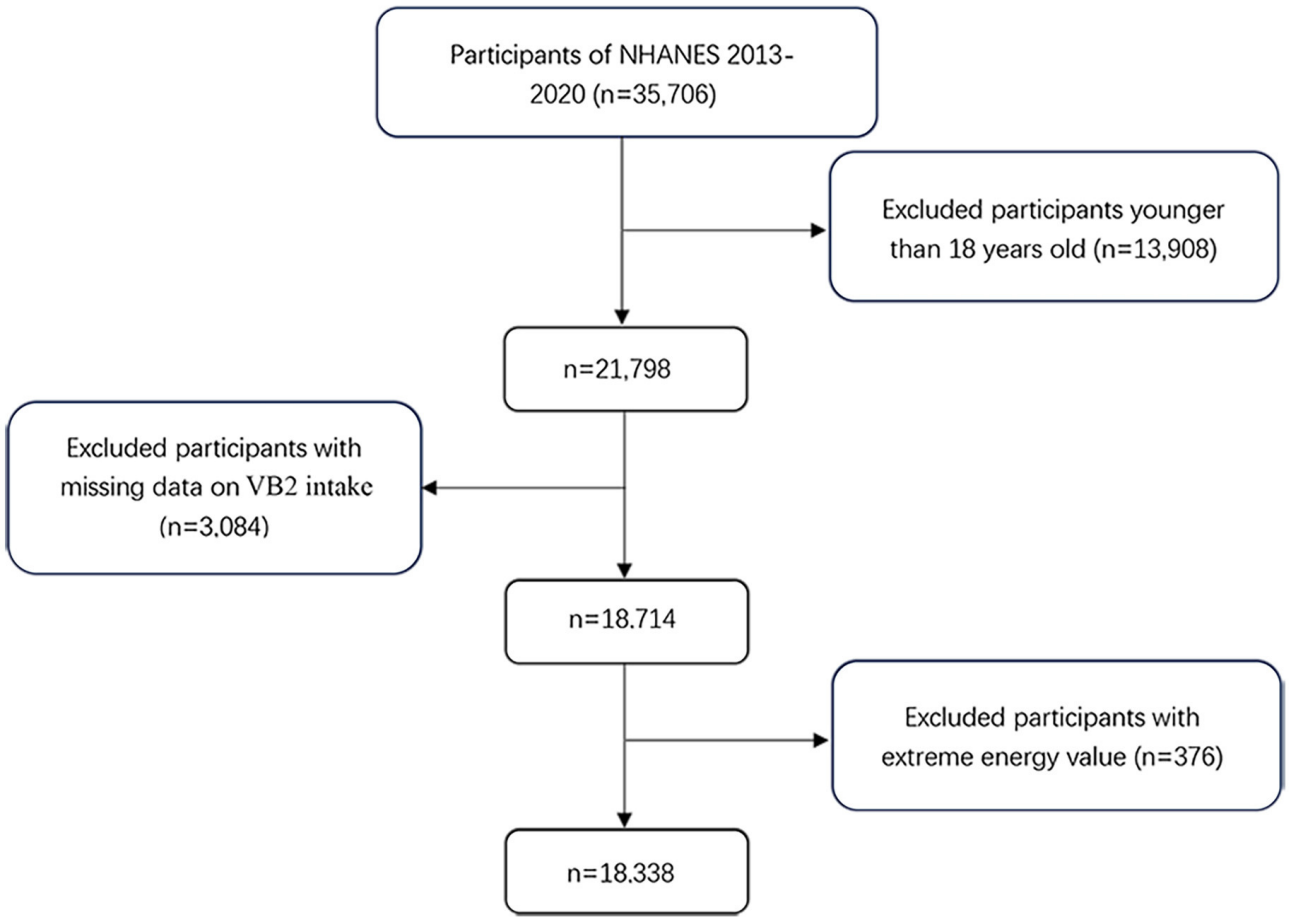
Flow chart of the study participants.

The NHANES is an ongoing nationally representative cross-sectional survey conducted in the United States every 2 years since early 1960s (21, 22). In the present study, we included four waves of the NHANES data in 2013–2014, 2015–2016, 2017–2018, and 2019–2020. Of the 35,506 participants, we excluded those under the age of 18 years (*n* = 13,908), with missing data on VB2 intake (*n* = 3,084) and key covariates (*n* = 376). Therefore, a total of 18,338 participants (8,869 men and 9,469 women) were included in the final analyses (Figure 1). The Ethics Review Board of the National Center for Health Statistics granted the posting of data online for public use, and all subjects provided written informed consent prior to study participation.

### 2.2 Assessment of VB2 intake

Data on VB2 intake including diet and supplement source, was collected via 24-h dietary recall (23). The first 24-h dietary recall was conducted personally at the mobile examination centers (MEC) and the second interview was over the phone 3–10 days later. We used the data from the first day to ensure accuracy. VB2 intake was calculated from the USDA's Food and Nutrient Dietary Study Database. Dietary supplement use was obtained by the questionnaire (24).

### 2.3 Definition of DM

Participants were defined as having DM if they met any of the following conditions: their fasting plasma glucose (FPG) ≥126 mg/dl (fasting 8–24 h); serum glucose at 2 h following a 75 g glucose load (oral glucose tolerance test) ≥200 mg/dl; glycohemoglobin>6.5%; self-reported DM in the NHANES questionnaire or use of glucose-lowering medicines (25, 26). We are not able to precisely decide the type of DM since the NHANES dataset does not provide enough information for such classification.

### 2.4 Covariates

In this study, we considered covariates including sex, age (18–44, 45–59, or ≥ 60 years), education level (less than high school, high school/GED, above high school), race (Mexican American, Other Hispanic, Non-Hispanic White, Non-Hispanic Black, Other race-including multi-racial), marital status (married and or partner/ widowed and divorced/never married), body mass index (BMI), energy intake (kcal/d, continuous), family poverty income ratio (PIR), self-reported hypertension and hyperlipidemia (27). BMI was calculated in the MEC by well-trained health technicians. Education level was assessed by the question “What is the highest grade or level of school you have completed or the highest degree you have received?”

### 2.5 Statistical analyses

Categorical variables were described as frequencies and percentages. And continuous variables were described as mean and standard deviation. Chi-square test was used to compare categorical data. VB2 intake was classified as sex-specific quartiles. We used weighted analysis to consider the complex sampling design of NHANES. Details can be found as follows: https://www.cdc.gov/nchs/ nhanes/index.htm. We used log transformation to normalize the distribution of VB-2 intake. Multivariable logistic regression analyses were conducted to evaluate the association between VB2 intake and DM. Results are shown as odds ratios (ORs) and 95% confidence interval (95% CI). Model 1 was adjusted for age and energy intake. Model 2 was further adjusted for race/ethnicity (Mexican American, other Hispanic, non-Hispanic White, non-Hispanic Black, or other Race-Including Multi-racial), marital status (married/partner, widowed/divorced, or never married), educational level (< high school, high school/GED, or >high school), PIR (< 1 or ≥1), BMI (< 25, 25–29.9, or ≥30 kg/m^2^). Model 3 was adjusted as for model 2 plus hypertension and hyperlipidemia. Tests for trend were performed by entering VB2 as a continuous variable in the regression model as a continuous variable and rerunning the corresponding regression model. Generalized additive model was used to examine potential non-linear association of VB2 intake with DM in women and men, separately.

We used stratified analyses to estimate potential effect modification by sex in the study population, and further by age, race/ethnicity, marital status, educational level, PIR, BMI, hypertension and hyperlipidemia in women. Tests of interactions were conducted by including a multiplicative term in the multivariable logistic models.

All statistical tests were two-sided and performed by SAS (version 9.4; SAS Institute, Inc., Cary, NC, USA) and R (“forestplot” package, version 4.5.0; R Foundation for Statistical Computing, Vienna, Austria). *P* < 0.05 was considered as statistically significant.

## 3 Results

### 3.1 Characteristics of the participants

Baseline characteristics of the participants are described in Table 1. Among the 18,338 participants, 3,455 had DM (percentage: 18.8%; 20.3% in men and 17.4% in women). In both women and men, compared with the participants without DM, those with DM were more likely to be older, widowed/divorced, have lower education, lower PIR, obesity, hypertension, and hyperlipidemia (*P* < 0.05). In addition, the women with DM had less VB2 intake than those without the condition (*P* = 0.017), whereas no significant difference in VB2 intake between the groups was observed in men.

**Table 1:** Baseline characteristics of the participants by diabetes (*n* = 18,338).

	**Women**			**Men**		
	**Without diabetes (*****n*** = **7,825)**	**Diabetes (*****n*** = **1,644)**	***P***	**Without diabetes (*****n*** = **7,066)**	**Diabetes (*****n*** = **1,803)**	***P***
**Age (years)**			< 0.001			< 0.001
18–44	3,710 (47.1)	260 (15.8)		3,383 (47.9)	187 (12.9)	
45–59	1,815 (26.2)	468 (28.5)		1,590 (22.5)	477 (32.7)	
≥60	2,285 (26.7)	916 (55.7)		2,093 (29.6)	1,134 (54.4)	
**Race/Ethnicity**			< 0.001			0.16
Mexican American	1,054 (13.5)	284 (17.3)		954 (13.5)	295 (16.4)	
Other Hispanic	863 (11.0)	211 (12.8)		704 (10.0)	180 (10.0)	
Non-Hispanic White	2,976 (38.0)	497 (30.2)		2,756 (39.0)	631 (35.0)	
Non-Hispanic Black	1,759 (22.5)	450 (27.4)		1,555 (22.0)	445 (24.6)	
Other race-including multi-racial	1,171 (15.0)	202 (12.3)		1,097 (15.5)	252 (14.0)	
**Marital status**			< 0.001			< 0.001
Married/partner	4,046 (53.6)	815 (49.8)		4,188 (61.6)	1,259 (70.0)	
Widowed/divorced	1,832 (24.3)	637 (38.9)		978 (14.4)	374 (20.8)	
Never married	1,671 (22.1)	186 (11.3)		1,504 (24.0)	163 (9.2)	
**Education level**			< 0.001			0.007
< High school	1,316 (17.2)	463 (29.4)		1,282 (19.4)	476 (26.6)	
High school/GED	1,695 (22.2)	410 (24.9)		1,605 (24.3)	428 (23.9)	
>High school	4,639 (60.6)	766 (46.7)		3,714 (56.3)	889 (49.5)	
**PIR**			0.11			0.05
< 1	2,359 (30.2)	557 (33.9)		1,963 (27.8)	497 (27.6)	
≥1	5,466 (69.8)	1,087 (66.1)		5,103 (72.2)	1,306 (72.4)	
**BMI (kg/m** ^2^ **)**			< 0.001			< 0.001
< 25	2,601 (33.2)	202 (12.3)		2,209 (31.2)	278 (15.4)	
25–29.9	2,169 (27.7)	369 (22.5)		2,597 (36.8)	607 (33.7)	
≥30	3,055 (39.1)	1,073(65.2)		2,260 (32.0)	918 (50.9)	
**Hypertension**			< 0.001			< 0.001
Yes	2,322 (29.7)	1,103(67.2)		2,010 (28.5)	1,162 (64.6)	
No	5,500 (70.3)	539 (32.8)		5,051 (71.5)	636 (35.4)	
**Hyperlipidemia**			< 0.001			< 0.001
Yes	2,130 (27.3)	970 (60.0)		2,103 (30.0)	1,037 (58.1)	
No	5,677 (72.7)	650 (40.0)		4,936 (70.0)	748 (41.9)	
**Smoking status**			< 0.001			< 0.001
Never	5,329 (68.1)	1,042 (63.4)		3,676 (52.0)	770 (42.7)	
Former	1,271 (16.2)	383 (23.3)		1,808 (25.6)	729 (40.4)	
Current	1,225 (15.7)	219 (13.3)		1,582 (22.4)	304 (16.9)	
Energy intake (kcal/d)	1,802.6 (709.2)	1,802.3 (679.6)	< 0.001	2,385.8 (1,136.2)	2,386.3 (981.7)	< 0.001
**Vitamin B2 intake (mg/day)**			0.017			0.41
Quartile 1	1,886 (24.1)	483 (29.4)		1,760 (24.9)	457 (19.1)	
Quartile 2	1,953 (24.9)	410 (24.9)		1,770 (25.0)	448 (21.8)	
Quartile 3	1,986 (25.4)	384 (23.4)		1,780 (25.2)	436 (25.3)	
Quartile 4	2,000 (25.6)	367 (22.3)		1,761 (24.9)	457 (33.8)	

All variables are expressed as frequencies and weighted percentages (%), except for energy intake (mean and standard deviation). P values were calculated using χ^2^ tests and t test. BMI, body mass index; GED, General Educational Development; PIR, ratio of family income to poverty.

BMI, body mass index; FPG, fasting plasma glucose; PIR, family poverty income ratio.

### 3.2 Association between VB2 intake and DM

**Figure 2:**
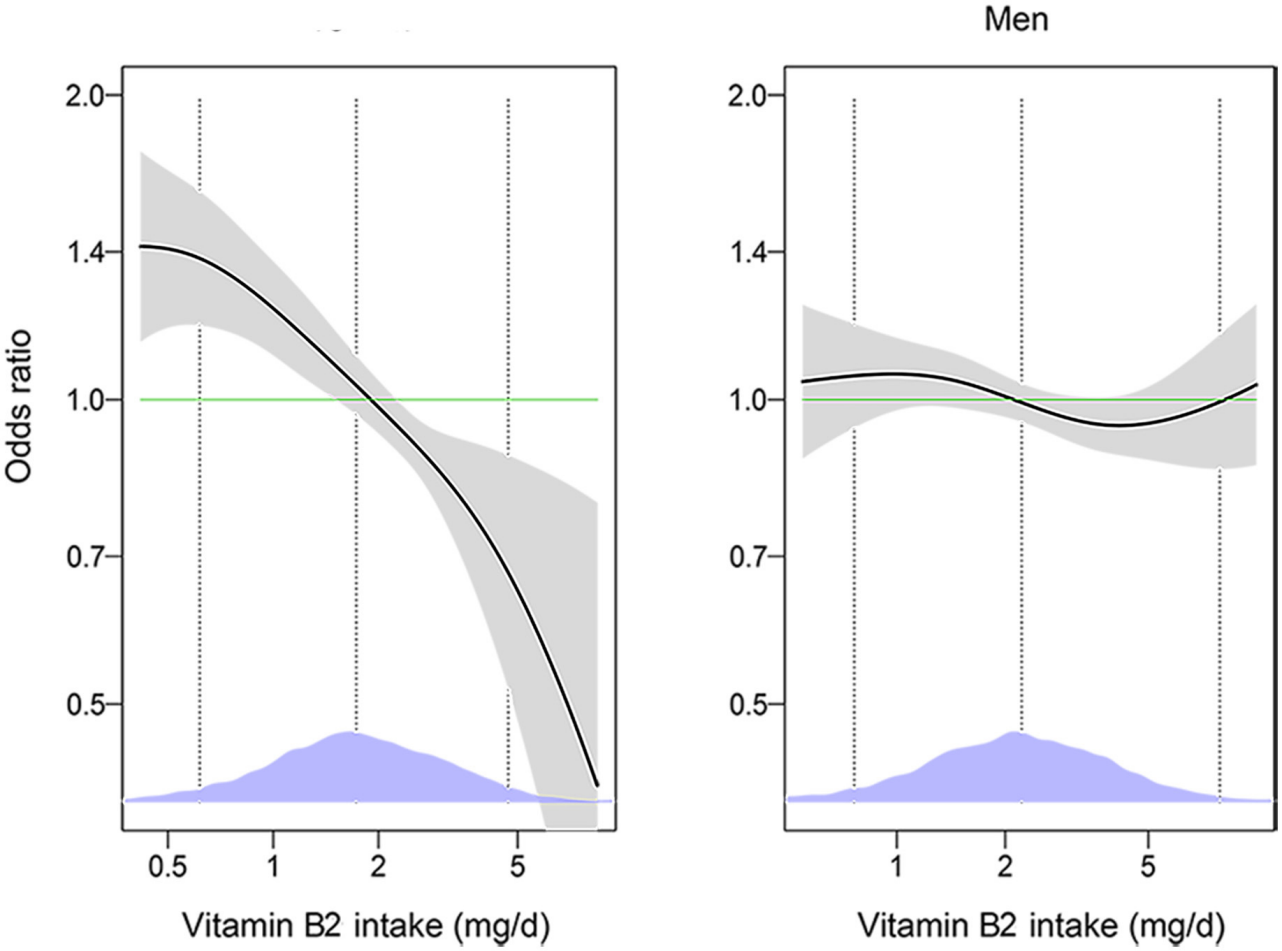
The associations of vitamin B2 with diabetes among men and women by generalized additive models (*n* = 18,338). The models were adjusted for age (18–44, 45–59, or ≥60 years), energy intake (continuous), race/ethnicity (Mexican American, other Hispanic, non-Hispanic White, non-Hispanic Black, or other Race-Including Multi-racial), marital status (married/partner, widowed/divorced, or never married), educational level (< high school, high school/GED, or >high school), PIR (< 1 or ≥1), BMI (< 25, 25–29.9, or ≥30 kg/m^2^), smoking status (every day, some days, or not at all), hypertension (yes or no), hyperlipidemia (yes or no). The solid lines show OR and the shaded areas 95% CI. The dashed lines show OR by linear regression on logarithmic scale. Density plots indicate the distributions of log-transformed vitamin B2 intake, and dotted lines denote the 10, 50, and 90th percentiles. BMI, body mass index; GED, General Educational Development; PIR, ratio of family income to poverty.

VB2 intake was significantly associated with DM in women but not in men (*P*-interaction < 0.05). As shown in Table 2, increased VB2 intake was significantly associated with lower odds of DM after adjustment for age and energy intake in women. The association remained significant after further adjustment for race/ethnicity, marital status, educational status, PIR, BMI and in the model with additional adjustment for hypertension and hyperlipidemia. The multivariable-adjusted OR for DM was 0.67 (95% CI: 0.48, 0.93) in the highest v. lowest quartiles of VB2 intakes, and 0.82 (95% CI: 0.71, 0.94) per 1- standard deviation (SD) increment of log-transformed VB2 intake. No significant association between VB2 intake and DM was observed in men. Consistently, generalized additive model showed that there was an inverse dose-response relationship between VB2 intake and DM in women, but not in men (Figure 2).

**Table 2:** Relationship between dietary vitamin B2 intake and diabetes by sex in the NHANES 2013–2020^a^.

	**Vitamin B2 intake (mg/day)**	**Case/*n***	**Model 1^b^**	**Model 2^c^**	**Model 3^d^**
Women	Quartile 1 (< 1.17)	483/2,369	1.00 (ref.)	1.00 (ref.)	1.00 (ref.)
	Quartile 2 (1.17–1.75)	410/2,363	0.66 (0.50, 0.87)	0.70 (0.52, 0.93)	0.69 (0.51, 0.93)
	Quartile 3 (1.76–2.70)	384/2,370	0.66 (0.52, 0.83)	0.73 (0.57, 0.93)	0.75 (0.57, 0.97)
	Quartile 4 (≥2.71)	367/2,367	0.59 (0.45, 0.77)	0.67 (0.49, 0.91)	0.67 (0.48, 0.92)
	*P* trend		< 0.001	0.013	0.025
	Continuous		0.78 (0.69, 0.88)	0.82 (0.71, 0.94)	0.81 (0.70, 0.94)
	*P*		< 0.001	0.004	0.005
Men	Quartile 1 (< 1.46)	457/2,217	1.00 (ref.)	1.00 (ref.)	1.00 (ref.)
	Quartile 2 (1.46–2.22)	448/2,218	0.80 (0.59, 1.07)	0.91 (0.71, 1.18)	0.90 (0.69, 1.18)
	Quartile 3 (2.23–3.39)	436/2,216	0.79 (0.62, 1.02)	0.95 (0.77, 1.18)	0.95 (0.75, 1.19)
	Quartile 4 (≥3.40)	457/2,218	0.93 (0.68, 1.27)	1.07 (0.81, 1.41)	1.03 (0.78, 1.35)
	*P* trend		0.184	0.503	0.672
	Continuous		1.01 (0.88, 1.17)	0.99 (0.89, 1.09)	1.00 (0.90, 1.10)
	*P*		0.85	0.79	0.96

^a^Logistic regression models were used to calculate the odds ratios and 95% confidence intervals.

^b^Model 1 was adjusted for age (18–44, 45–59, or ≥ 60 years) and energy intake (continuous).

^c^Model 2 was adjusted as for model 1 plus race/ethnicity (Mexican American, other Hispanic, non-Hispanic White, non-Hispanic Black, or other race-including multi-racial), marital status (married/partner, widowed/divorced, or never married), educational level (< high school, high school/GED, or >high school), PIR (< 1 or ≥1), BMI (< 25, 25–29.9, or ≥30 kg/m^2^).

^d^Model 3 was adjusted as for model 2 plus hypertension (yes or no), and hyperlipidemia (yes or no).

### 3.3. Stratified analyses

**Figure 3:**
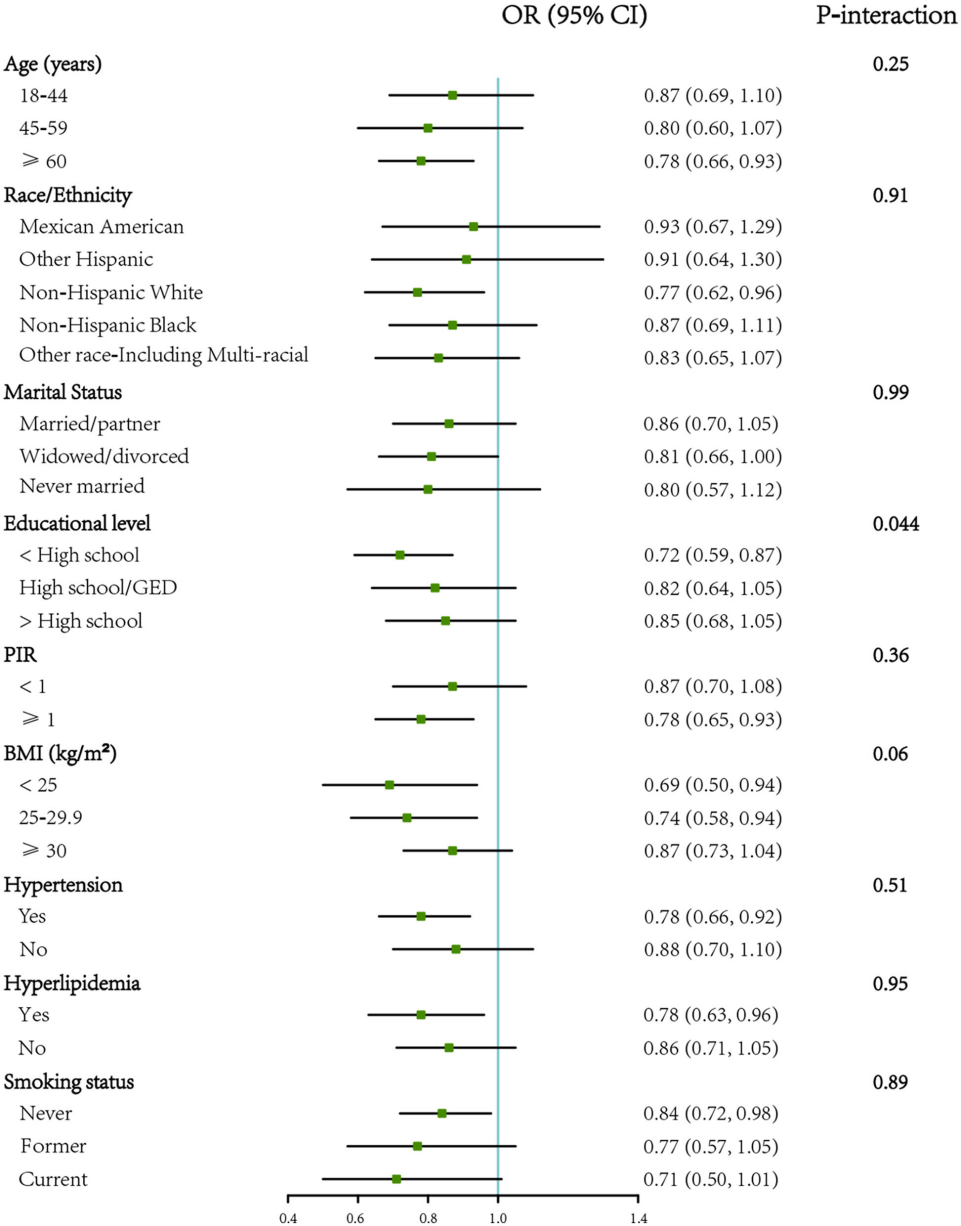
Forest plot showing the associations of VB2 intake with diabetes by different characteristics in women (*n* = 9,469). OR (95% CI) are reported per 1-SD increment of log-transformed vitamin B2 intake. The models were adjusted for age (18–44, 45–59, or ≥60 years), energy intake (continuous), race/ethnicity (Mexican American, other Hispanic, non-Hispanic White, non-Hispanic Black, or other Race-Including Multi-racial), marital status (married/partner, widowed/divorced, or never married), educational level (<high school, high school/GED, or >high school), PIR (<1 or ≥1), BMI (<25, 25–29.9, or ≥30 kg/m^2^), hypertension (yes or no), hyperlipidemia. BMI, body mass index; GED, General Educational Development; PIR, ratio of family income to poverty. SD, standard deviation.

In women, the association between VB2 intake and DM was similar in subgroups stratified by age, race/ ethnicity, marital status, PIR, BMI, hypertension and hyperlipidemia (*P* for interaction > 0.05), except education level (*P* for interaction = 0.044). The association of VB2 intake with DM was more prominent among those with an education level lower than high school (Figure 3).

## 4 Discussion

In this large cross-sectional study, we observed that higher VB2 intake was strongly associated with lower odds of DM in women, but not in men. The inverse association in women was independent of potential confounders and similar across subgroups except for educational level.

Our findings were supported by a prospective study from Japan, which reported that dietary VB2 intake was inversely associated with the risk of DM only in women aged 40–79 years (18). A cohort study in the U.S. also suggested that high intake of VB2, especially from food sources, may be associated with a lower risk of DM in both women and men (14). However, the study population of this study unrepresentative of the U.S. population.

We observed a significant interaction between VB2 intake and education levels in association with DM among women. The reason for this may be that highly educated women prefer a nutritionally balanced diet, which masked the potential protective effect of VB2 intake in this population (28).

VB2 is a core component of the cofactors flavin adenine dinucleotide (FAD) and flavin mononucleotide (FMN) (29). FMN and FAD play important roles in energy metabolism as two forms of VB2 in cells. Oxidative stress is the result of an imbalance between the production of free radicals and the ability to deactivate them, which increases the risk of DM (12). Riboflavin can act as an antioxidant by scavenging free radicals (30). Oxidative stress properties play a crucial role in the pathogenesis of cardiovascular diseases (31) such as DM (32, 33). An animal study also suggested that riboflavin can serve as an antioxidant against antioxidant stress, especially lipid peroxidation and oxidative DNA damage (34). VB2 may decrease risk of DM through reduced inflammation, oxidative stress, and hyperglycemia (17). We observed the inverse association only in women. Men may differ from women in their dietary habits and consume fewer VB2 supplements (35). Maybe dietary and supplement intake of VB2 is not sufficient to offset the risk associated with poor lifestyle in men, and therefore VB2 intake was not associated with DM in men (18). We also speculate that women may need more VB2 during special physiological stages of menstruation, pregnancy, and lactation, resulting in a relative deficiency of VB2 (36). Moreover, some sex variations in hormones, body composition, and fat metabolism may account for the observed effect modification (37). At the time of diagnosis of type 2 DM, women typically show a higher burden of risk factors than men, including higher blood pressure and greater excess weight gain (38). Some studies indicated that premenopausal women may have higher insulin sensitivity to skeletal muscle and liver, and higher stimulated insulin secretion (37).

Our study has the following strengths. First, the present study had a nationally representative sample and a large sample size by combining data from four waves of the NHANES survey. Therefore, our results could be generalized to the whole U.S. population. Second, we controlled for a number of confounding factors including socio-demographic, health and lifestyle information so that we can better estimate the association between VB2 intake and DM. However, there are also several limitations of this study. First, since our study was based on a cross-sectional design, a causal relationship between VB2 intake and risk of DM could not be inferred. Second, we used only the first day's dietary recall data in the present study, which could not avoid random measurement errors. Third, this study was conducted in the U.S. and may not be generalizable to other populations.

## 5 Conclusions

In summary, our study showed a significantly inverse association between VB2 intake and DM in U.S. women, but this association was not present in men. Further evidence from other prospective cohort studies is needed to confirm our findings.

## Data Availability

The datasets presented in this study can be found in online repositories. The names of the repository/repositories and accession number(s) can be found at: www.cdc.gov/nchs/nhanes.
